# Interplay between H3K36me3, methyltransferase SETD2, and mismatch recognition protein MutSα facilitates processing of oxidative DNA damage in human cells

**DOI:** 10.1016/j.jbc.2022.102102

**Published:** 2022-06-03

**Authors:** Sida Guo, Jun Fang, Weizhi Xu, Janice Ortega, Chang-Yi Liu, Liya Gu, Zhijie Chang, Guo-Min Li

**Affiliations:** 1 Tsinghua-Peking Center for Life Sciences and Department of Basic Medical Sciences, Tsinghua University, Beijing, China; 2 Department of Radiation Oncology, University of Texas Southwestern Medical Center, Dallas, Texas, USA; 3 Department of Cardiology, Peking Union Medical College, Chinese Academy of Medical Sciences, Beijing, China

**Keywords:** oxidative DNA damage, SETD2, histone methylation, mismatch repair, ChIP-Seq, chromatin immunoprecipitation assays combined with DNA sequencing, Co-IP, coimmunoprecipitation, DDR, DNA damage response, FACS, fluorescence-activated cell sorting, KI, knock-in, MMR, mismatch repair, RIPA, radioimmunoprecipitation assay, ROS, reactive oxygen species, TSS, transcription start site

## Abstract

Oxidative DNA damage contributes to aging and the pathogenesis of numerous human diseases including cancer. 8-hydroxyguanine (8-oxoG) is the major product of oxidative DNA lesions. Although OGG1-mediated base excision repair is the primary mechanism for 8-oxoG removal, DNA mismatch repair has also been implicated in processing oxidative DNA damage. However, the mechanism of the latter is not fully understood. Here, we treated human cells defective in various 8-oxoG repair factors with H_2_O_2_ and performed biochemical, live cell imaging, and chromatin immunoprecipitation sequencing analyses to determine their response to the treatment. We show that the mismatch repair processing of oxidative DNA damage involves cohesive interactions between mismatch recognition protein MutSα, histone mark H3K36me3, and H3K36 trimethyltransferase SETD2, which activates the ATM DNA damage signaling pathway. We found that cells depleted of MutSα or SETD2 accumulate 8-oxoG adducts and fail to trigger H_2_O_2_-induced ATM activation. Furthermore, we show that SETD2 physically interacts with both MutSα and ATM, which suggests a role for SETD2 in transducing DNA damage signals from lesion-bound MutSα to ATM. Consistently, MutSα and SETD2 are highly coenriched at oxidative damage sites. The data presented here support a model wherein MutSα, SETD2, ATM, and H3K36me3 constitute a positive feedback loop to help cells cope with oxidative DNA damage.

Exposure to ionizing radiation or reactive oxygen species (ROS) results in oxidative stress and the formation of large amounts of oxidative DNA lesion 7,8-dihydro-8-oxoguanine (8-oxoG) (1, 2), which contributes to aging and can lead to human diseases like cancer and neurological disorders (3, 4, 5, 6, 7). In response to oxidative stress, cells activate DNA damage response and repair pathways. 8-oxoG DNA glycosylase 1 (OGG1)–mediated base excision repair is the primary mechanism responsible for excising 8-oxoG from DNA (2, 8). However, there is a slow but significant removal of 8-oxoG in *Ogg1*-KO cells (9), which suggests an additional mechanism(s) for 8-oxoG removal (9). The DNA mismatch repair (MMR) pathway has been implicated in processing oxidative DNA lesions (10, 11, 12, 13, 14).

MMR is known for maintaining replication fidelity by correcting biosynthetic errors generated during DNA replication (15, 16, 17, 18). Defects in MMR can lead to cancer development and bolster cancer cell resistance to many chemical and physical agents (17, 19). The mismatch recognition protein MutSα, a key MMR factor consisting of the MSH2 and MSH6 subunits, identifies mismatches and recruits downstream factors to trigger mismatch-provoked incision and exonuclease 1-catalyzed mismatch removal, followed by DNA polymerase δ–conducted DNA repair synthesis (20, 21). However, MutSα's participation in MMR in human cells relies on histone H3 lysine 36 trimethylation (H3K36me3) (22), an important histone mark that is highly enriched in gene bodies and actively transcribed regions (23). MutSα's MSH6 subunit contains a PWWP domain and can specifically interact with H3K36me3. This interaction recruits MutSα to replicating chromatin (22). Thus, factors that regulate H3K36me3 levels (*e.g.*, histone methyltransferases and histone demethylases) and/or the H3K36me3–MutSα interaction are expected to influence MMR activity (24). Indeed, genetic defects in the H3K36 trimethyltransferase gene *SETD2* (25, 26, 27) impair the MMR function (22), and histone mutations that disrupt H3K36me3’s interaction with SETD2 or MutSα result in MMR deficiency (22, 23, 28). Interestingly, although MMR is coupled with replication (29, 30), emerging evidence suggests that it also maintains genome stability during transcription (23, 31). We have shown that the localization of MutSα in actively transcribed genes by H3K36me3 is essential for protecting these genes from mutation and that disrupting the H3K36me3–MutSα interaction preferentially induces mutations in actively transcribed genes when cells are treated with H_2_O_2_ (23). Cells defective in MutSα are more sensitive to oxidative stress than MMR-proficient cells (32, 33). However, the molecular mechanism by which the MMR system processes oxidative DNA damage is unclear.

In addition to recognizing mismatches, MutSα also recognizes many nonmismatch DNA lesions, including 8-oxoG (11, 12, 34). MutSα's recognition of nonmismatch DNA lesions can trigger the DNA damage response (DDR) (17, 19). Both the ataxia-telangiectasia mutated (ATM) and ATM and rad3-related (ATR) signaling pathways are involved in this process, though the former is specific to DNA lesions induced by ionizing radiation (35), which also generates ROS (36), and the latter appears to deal with alkylating DNA adducts (37, 38). SETD2, also known as KMT3 and HYPB, is required for ATM-dependent and p53-mediated checkpoint in response to DNA damage (39). Interestingly, ATM has been shown to act as an important sensor of ROS in human cells, as it targets on a large number of protein substrates in response to oxidative stress (40). We hypothesize that ATM is part of the MutSα-SETD2-H3K36me3 signaling pathway in response to oxidative DNA damage.

In this study, we analyzed cellular responses to H_2_O_2_ in cells with various MMR activities. We show that cells depleted of MutSα, SETD2, or H3K36me3 accumulate 8-oxoG adducts and are more sensitive to H_2_O_2_ than WT cells. MutSα, SETD2, and ATM collaborate with each other to process oxidative DNA damage. SETD2 interacts with both MutSα and ATM through its SET domain. Upon H_2_O_2_ treatment, MutSα and SETD2 are highly coenriched in promoter regions/transcription start sites (TSSs), and both are essential for activating the ATM signaling pathway. Our data presented here support a model where MutSα, SETD2, ATM, and H3K36me3 constitute a positive feedback loop to cope with oxidative DNA damage.

## Results

### MutSα is enriched in chromatin upon H_2_O_2_ treatment

**Figure 1:**
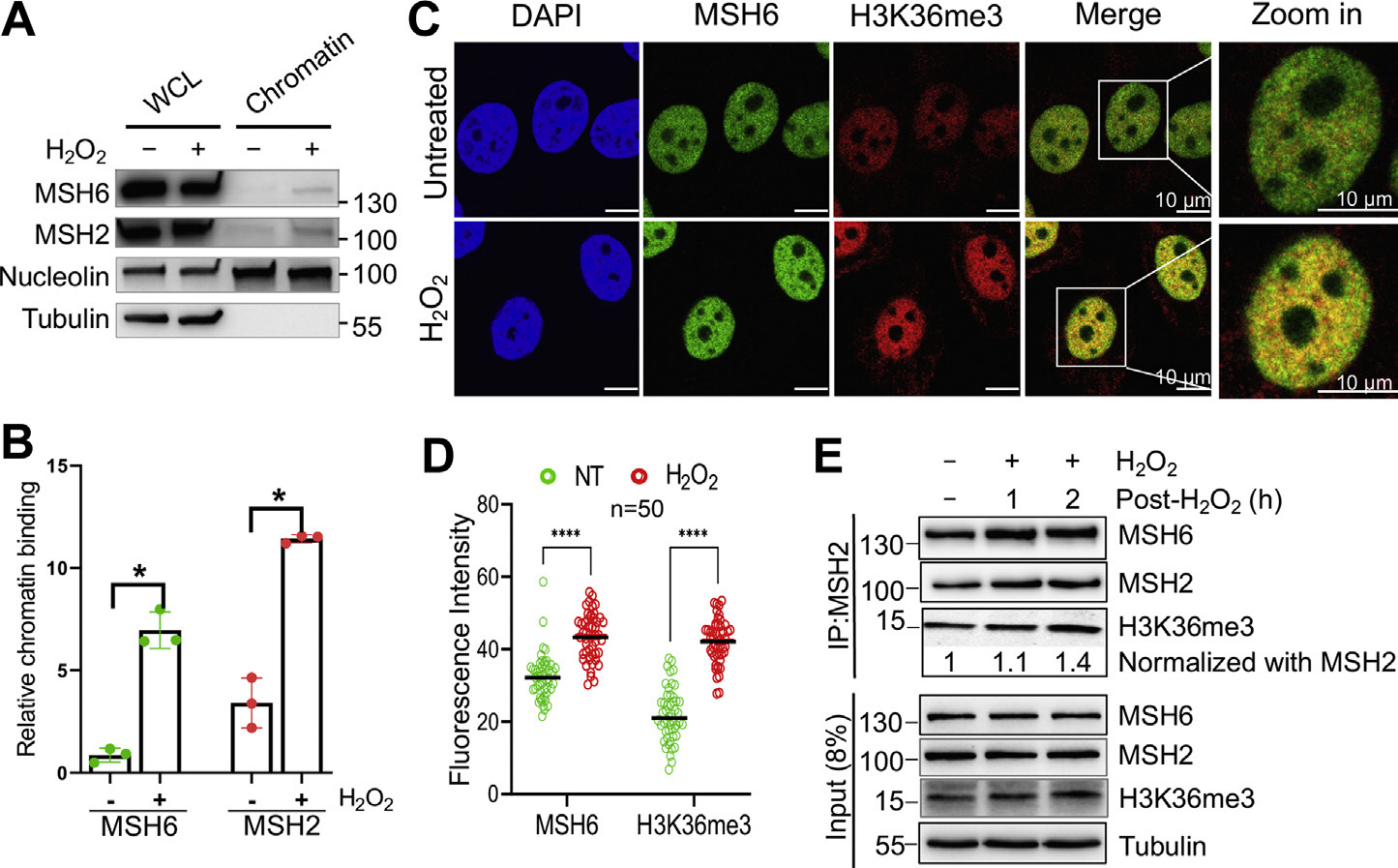
**Enhanced recruitment of MutSα to chromatin upon H**_**2**_**O**_**2**_**treatment.***A*, Western blots showing increased chromatin binding of MutSα by H_2_O_2_. Nucleolin and tubulin were used as non-H_2_O_2_-specific controls. *B*, statistical analysis of relative amounts of chromatin-bound MutSα, as shown in (*A*). Data were derived from three independent determinants. Error bars represent mean ± SEM. ∗ indicates *p* < 0.05 (two-tailed *t* test). *C*, representative immunofluorescence images showing enhanced chromatin binding of MSH6 and enrichment of H3K36me3 by H_2_O_2_. *D*, quantification of fluorescence intensity of MSH6 and H3K36me3 in response to H_2_O_2_ treatment. NT, untreated. *E*, Co-IP Western analysis to detect H_2_O_2_-induced increase in the interaction between H3K36me3 and MSH6. Co-IP, coimmunoprecipitation; WCL, whole cell lysate.

To determine how MMR deals with oxidative DNA damage, we treated HeLa cells with 1 mM of H_2_O_2_ for 30 min, a condition that allows ∼80% surviving rate in clonogenic analysis. We first determined the MutSα (MSH2-MSH6) level in the chromatin fraction of cells treated with or without H_2_O_2_. Although the total cellular protein levels of MutSα between H_2_O_2_-treated and untreated cells were about the same, the level of chromatin-bound MutSα was significantly higher in treated cells than in untreated cells (Fig. 1, *A* and *B*). We then performed confocal immunofluorescence microscopy analysis to directly visualize MutSα recruitment to chromatin by H3K36me3. The results show that H_2_O_2_ treatment enhanced the enrichment of both H3K36me3 and MSH6 on chromatin, which significantly increased the colocalization of H3K36me3 and MSH6 (Fig. 1, *C* and *D*). These results suggest that H_2_O_2_ treatment promotes the production of H3K36me3, which in turn recruits more MutSα to chromatin, as previously demonstrated (22). Coimmunoprecipitation (Co-IP) using an MSH2 antibody confirmed this conclusion, as more MSH6 and H3K36me3 were coprecipitated in H_2_O_2_-treated cells than in controls after normalization with the MSH2 levels in corresponding reactions (Fig. 1*E*). Taken together, these observations indicate that oxidative DNA damage enriches the production of H3K36me3, thereby efficiently recruiting MutSα to chromatin.

### ATM activation is associated with MutSα-mediated response to oxidative stress

**Figure 2:**
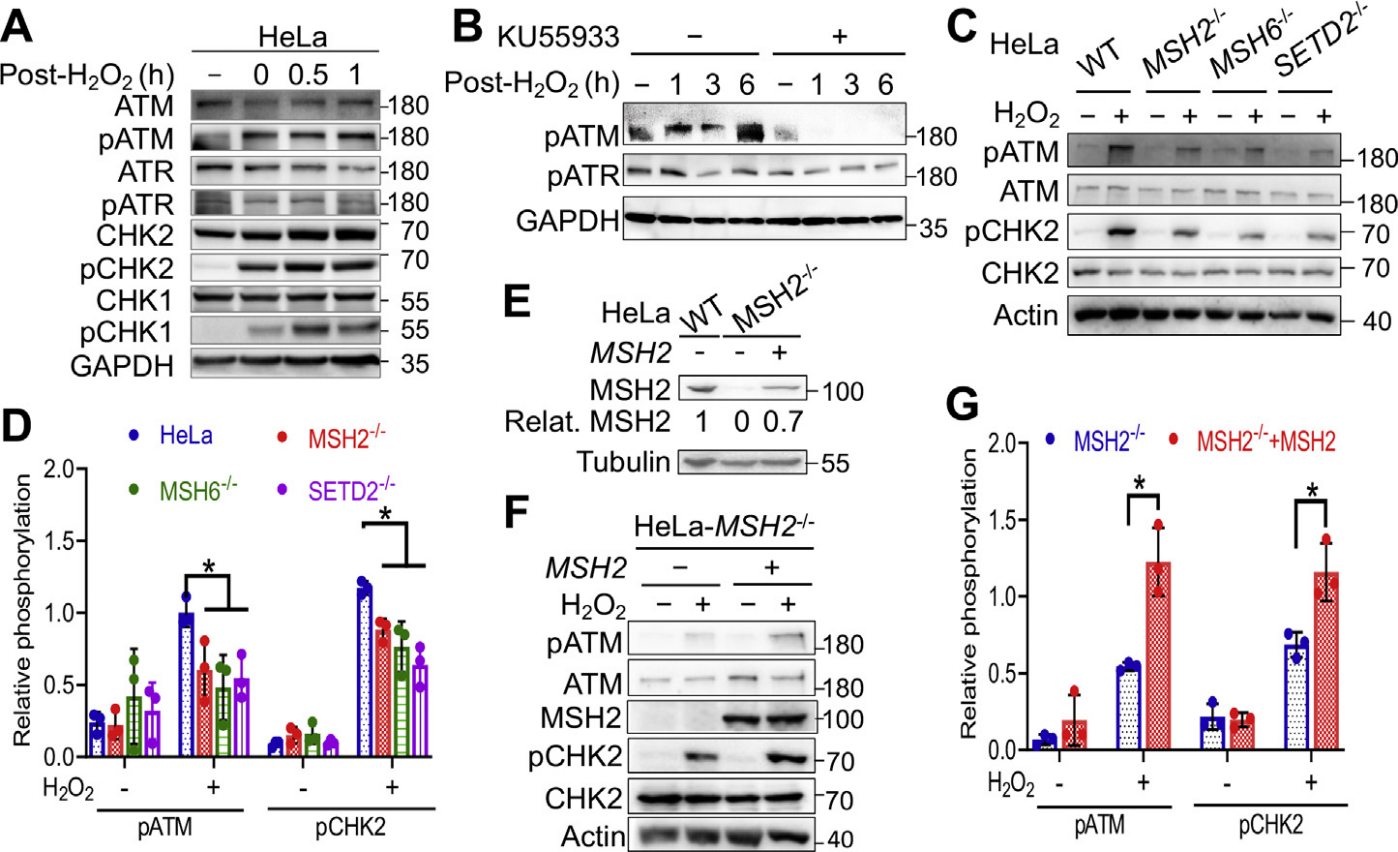
**MMR-mediated oxidative response activates the ATM signaling pathway.***A*, Western blots showing H_2_O_2_-induced phosphorylation of ATM but not ATR. *B*, Western blots showing inhibition of ATM phosphorylation by KU55933 (15 µM). *C*, Western blot analysis showing reduced H_2_O_2_-induced phosphorylation of ATM and CHK2 in cells depleted of MSH2, MSH6, or SETD2. *D*, quantification of Western blots, as shown in (*C*). Data were derived from three independent determinants. Error bars represent mean ± SEM. ∗ indicates *p* < 0.05 (two-tailed *t* test). *E*, Western blot analysis to show the *MSH2* expression level in the rescue experiment. *F*, Western blotting assays showing MutSα-dependent ATM activation by H_2_O_2_. *G*, statistical analysis of Western blotting data shown in (*F*). Data were derived from three independent determinants. Error bars represent mean ± SEM. ∗ indicates *p* < 0.05 (two-tailed *t* test). MMR, mismatch repair.

ATR and ATM have been implicated in MMR-mediated DNA damage signaling in response to alkylating agents (37, 38) and ionizing radiation (35), respectively. To determine whether ATM or ATR is involved in oxidative stress–induced DDR, we measured the activation of these two molecules at different time points after H_2_O_2_ treatment. We observed ATM phosphorylation immediately following H_2_O_2_ treatment but detected background levels of phosphorylated ATR (Fig. 2*A*), which suggests that ATM, but not ATR, participates in oxidative stress–induced DDR. Consistent with ATM activation, we detected the phosphorylation of CHK2 kinase (Fig. 2*A*), a downstream substrate of the ATM kinase. However, although ATR is not activated, its downstream substrate CHK1 kinase is phosphorylated (Fig. 2*A*), which is probably due to the overlapping but nonredundant activities with substantial crosstalk between the ATM and ATR pathways (41) or an ATR-independent protein kinase (42). To further confirm that H_2_O_2_-induced DDR is mediated through ATM but not ATR signaling, we analyzed the phosphorylation status of these two protein kinases in cells treated with or without ATM kinase–specific inhibitor KU55933 (43). We found that H_2_O_2_-induced ATM phosphorylation was completely inhibited by KU55933, but the phosphorylation status of ATR was essentially unaffected regardless of H_2_O_2_ treatment (Fig. 2*B*).

To determine the role of MutSα in H_2_O_2_-induced ATM signaling, we knocked out *MSH2*, *MSH6*, or *SETD2* in HeLa cells and analyzed the resulting KOs, *MSH2*^*−/−*^, *MSH6*^*−/−*^, and *SETD2*^*−/−*^, for ATM signaling in response to H_2_O_2_ treatment. The results revealed that each of the KO cells exhibited lower levels of ATM phosphorylation than WT cells, and the same was also true for CHK2 phosphorylation (Fig. 2*C*). The reduction in ATM and CHK2 phosphorylation levels in KO cells was significant (Fig. 2*D*). Restoring MMR function in these MMR-KO cells also restores their H_2_O_2_-induced ATM signaling. For example, when MSH2 expression (70% of the native level) was restored in HeLa-MSH2^−/−^ cells (Fig. 2*E*), significantly higher levels of ATM/CHK2 phosphorylation were detected (Fig. 2, *F* and *G*). These results suggest that H_2_O_2_-induced ATM signaling depends on MutSα.

### MutSα, SETD2, and ATM process 8-oxoG in the same pathway

**Figure 3:**
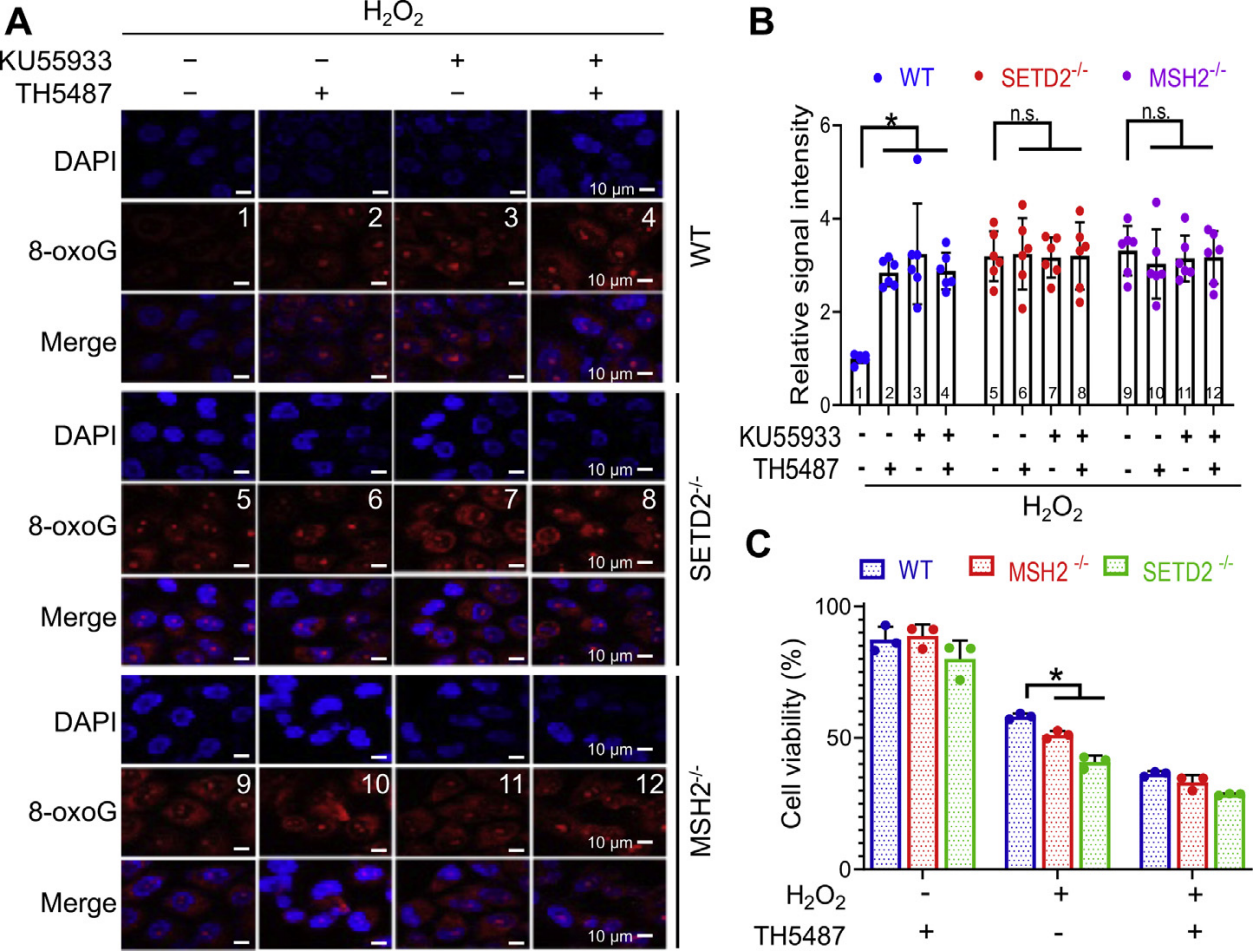
**MutSα and SETD2 process 8-oxoG adducts independently of OGG1.***A*, visualization of 8-oxoG levels in WT, *MSH2*^*−/−*^, and *SETD2*^*−/−*^ HeLa cells by immunostaining. Ku-55933 (15 µM)  and TH5487 (10 µM) are ATM and OGG1 inhibitors, respectively. *B*, quantification of 8-oxoG levels shown in (*A*). Data show the mean ± SEM of 8-oxoG relative intensity from six nuclei/treatment. ∗ indicates *p* < 0.05 (two-tailed *t* test). *C*, cell survival assay by FITC-annexin V and PI double staining, followed by flow cytometry. Data were derived from three independent determinants. ∗ indicates *p* < 0.05 (two-tailed *t* test).

To determine the relationship between MutSα, SETD2, and ATM in the cellular response to oxidative stress, we used an 8-oxoG-specific antibody to detect 8-oxoG levels in cells with or without functional MMR, SETD2, or ATM after H_2_O_2_ treatment. We first determined whether the MutSα/SETD2/ATM–mediated oxidative response depends on OGG1, the primary enzyme responsible for 8-oxoG removal (2, 8). As expected, HeLa cells treated with an OGG1 inhibitor, TH5487, exhibited a significantly higher 8-oxoG level than untreated cells (Fig. 3, *A* and *B*, compare treatments 1 and 2). The same analysis was performed in cells that are proficient in OGG1 but depleted of MSH2, SETD, or ATM. The results showed that MSH2 KO (*MSH2*^*−/−*^) cells contained higher levels of 8-oxoG adducts than WT cells (Fig. 3, *A* and *B*, compare treatments 1 and 9), which is consistent with the fact that MMR processes 8-oxoG in an OGG1-independent manner (14). The same results were also observed in *SETD2*^*−/−*^ cells (Fig. 3, *A* and *B*, compare treatments 1 and 5), which indicates that SETD2 is involved in 8-oxoG removal. Since SETD2 is responsible for the production of H3K36me3 to recruit MutSα to chromatin (22), SETD2’s role in processing 8-oxoG may go through the MMR pathway. Finally, we measured 8-oxoG levels in cells cultured in the presence of KU55933, an inhibitor specifically for the ATM kinase (43), and found that, like *MSH2*^*−/−*^ and *SETD2*^*−/−*^ cells, WT cells treated with KU55933 displayed a significantly higher 8-oxoG level than untreated cells (Fig. 3*A*, compare treatments 1 and 3). Interestingly, we did not observe a further increase in 8-oxoG levels when *SETD2*^*−/−*^ or *MSH2*^*−/−*^ cells were treated with KU55933 (Fig. 3, *A* and *B*, compare treatment 5 with treatment 7, treatment 9 with 11). This suggests that MutSα, SETD2, and ATM process oxidative DNA damage in the same pathway. Similarly, we also did not observe an increase in 8-oxoG levels in *SETD2*^*−/−*^ or *MSH2*^*−/−*^ cells when they were treated with the OGG1 inhibitor TH5487 (Fig. 3, *A* and *B*, compare treatments 5 and 6, treatments 9 and 10). This is probably due to that *SETD2*^*−/−*^ or *MSH2*^*−/−*^ cells accumulating high levels of oxidative DNA damage did not survive the treatment (see later and the data shown in Fig. 3*C*) and thus were uncollectible in this analysis.

It is well known that, in response to DNA damage, cellular fate is determined by the DNA repair machinery’s ability to restore DNA integrity. If DNA damage is left unrepaired, the cell death program will be activated (44, 45). To test the impact of MMR on cell fate in response to oxidative stress in the absence of OGG1, we treated *MSH2*^*−/−*^ or *SETD2*^*−/−*^ cells with H_2_O_2_ for 30 min and cultured them in fresh medium for 24 h before harvesting for fluorescence-activated cell sorting (FACS) analysis to determine cell viability. The results showed that almost all H_2_O_2_-untreated cells, regardless of MMR background (WT, *MSH2*^*−/−*^ or *SETD2*^*−/−*^), were viable upon FACS analysis, even though their OGG1 activity was blocked (Fig. 3*C*). However, H_2_O_2_ treatment greatly reduced cell viability, particularly in *MSH2*^*−/−*^ and *SETD2*^*−/−*^ cells, and inhibiting OGG1 further enhanced cell sensitivity to H_2_O_2_ (Fig. 3*C*). These results suggest that MMR processing of oxidative DNA damage is important for genome stability and cell survival.

It is worth mentioning that *MSH2*- or *SETD2*-deficient cells display no additional sensitivity to H_2_O_2_ when OGG1 is inhibited, as compared with WT cells (Fig. 3, the middle group). It is known that MMR-deficient cells are more tolerant to chemicals such as cisplatin and methylating agents than MMR-proficient cells (19, 46). This is because the MMR system recognizes and processes the chemically modified DNA lesions *via* the so-called futile repair pathway, where the offending DNA adducts located in the template DNA strand constantly trigger the MMR reaction, which only targets the newly synthesized strand for mismatch removal. Ultimately, this futile repair cycle induces apoptosis (19, 47). However, MMR-deficient cells fail to initiate the repair process to move the DNA lesions. Thus, despite accumulating numerous mutations, they survive chemical treatments. It is possible that inhibition of OGG1 may have adapted the resistant nature of MMR-deficient cells during H_2_O_2_ treatment. The futile repair theory may also explain why there is essentially no difference in the cell survival rate between WT and MMR-deficient cells when they were treated with H_2_O_2_ in the presence of TH5487 (Fig. 3*C*, the right group), as 8-oxoG adducts can induce apoptosis in WT, but not in mutant cells. Thus, the 8-oxoG-provoked apoptosis in WT cells erases their growth advantage over the H_2_O_2_-sensitive MMR-deficient cells when OGG1 is inhibited. Future studies will further elucidate the molecular details.

### SETD2 interacts with MutSα and ATM *via* its SET domain

**Figure 4:**
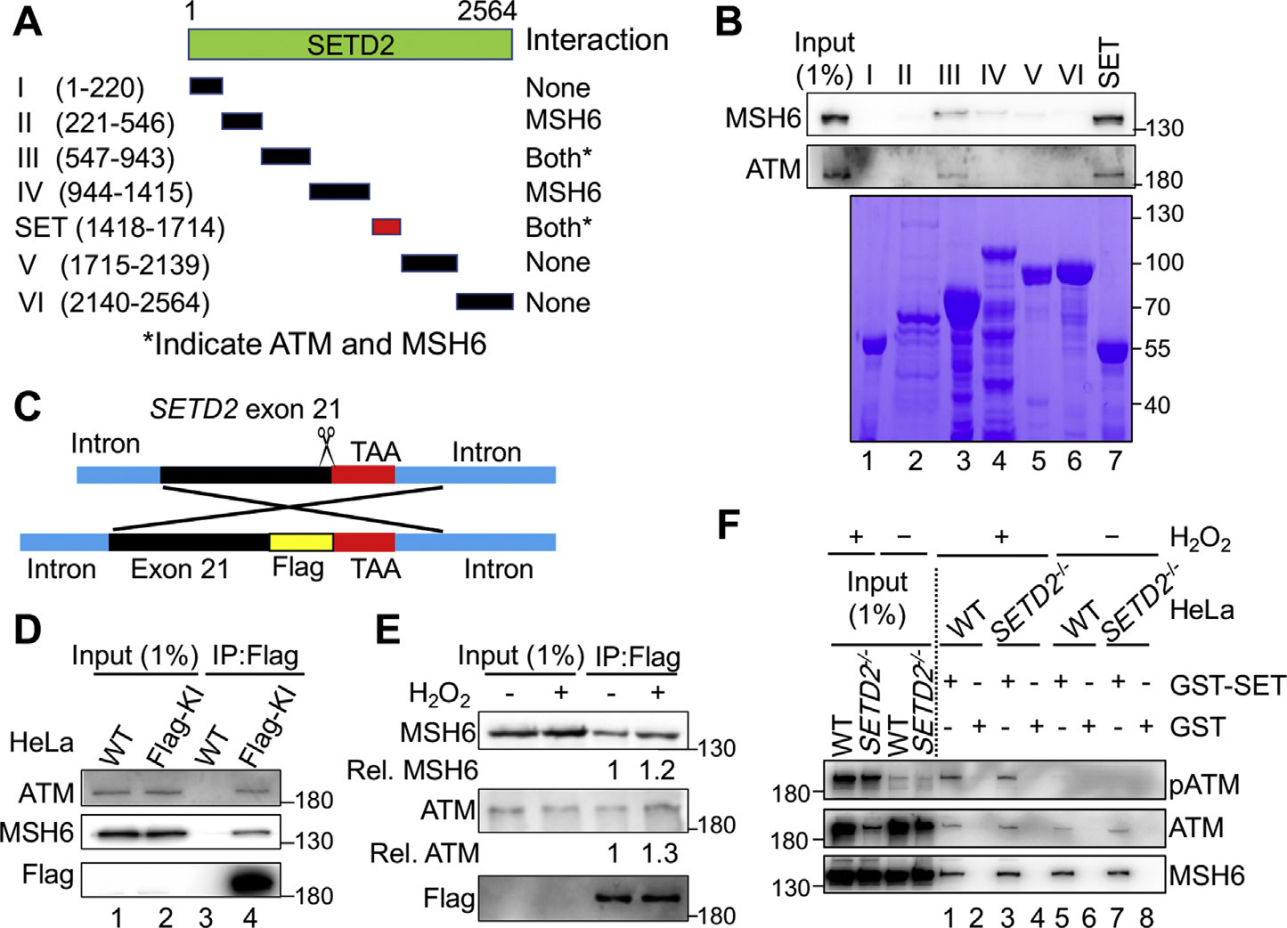
**SETD2 interacts with MutSα and ATM through its SET domain.***A*, schematic diagram of the individual SETD2 fragments used in the GST pull-down assay. *B*, GST pull-down assay showing the physical interaction of SETD2 with MSH6 and ATM *via* the SET domain. The individual GST-SETD2 fragments are shown by Coomassie blue staining (*bottom*). *C*, schematic diagram illustrating the strategy of FLAG-tag knock-in right before the stop codon of the *SETD2* gene by the CRISPR-Cas9 system to generate a C-terminal FLAG-tagged SETD2 protein. *D* and *E*, Co-IP analysis to determine FLAG-SETD2’s interaction with ATM and MSH6 in the absence (*D*) or presence (*E*) of H_2_O_2_ treatment. *F*, immunoblots showing pull down of MSH6 and ATM by GST-SET in H_2_O_2_-treated and untreated cells; pATM was only pulled down in H_2_O_2_-treated cells. Co-IP, coimmunoprecipitation.

The aforementioned findings prompted us to hypothesize that MutSα, SETD2, and ATM interact with each other in response to oxidative DNA damage. To test this hypothesis, we generated seven glutathione S-transferase (GST)-tagged SETD2 fragments, including the SET domain (aa 1418–1714), which is the enzymatic motif responsible for trimethylating H3K36 (Fig. 4*A*). We used the resulting SETD2 fragments to pull down ATM and MutSα in HeLa cell lysates. Both MSH6 (*i.e.*, MutSα) and ATM interacted with the SET domain of SETD2 (Fig. 4*B*, *lane 7*). In addition, both proteins showed a weaker interaction with fragment III (*lane 3*). These observations suggest that both MutSα and ATM physically interact with SETD2 *via* the same SETD2 domain.

We then conducted Co-IP analysis to verify SETD2’s interactions with MutSα and ATM. However, because of lacking a quality SETD2 antibody, we failed to pull down and specifically detect SETD2 in Co-IP assays. To solve this problem, we knocked-in the sequence coding for the FLAG tag (DYKDDDDK) in the C terminus of SETD2 *via* CRISPR-Cas9 technology (Fig. 4*C*). The resulting FLAG knock-in (KI) HeLa cells were confirmed by DNA sequencing and used to determine these protein–protein interactions by Co-IP. The results showed that both MSH6 and ATM were coprecipitated with FLAG-tagged SETD2 in cell lysates derived from FLAG-KI HeLa cells (Fig. 4*D*, *lane 4*), but not in lysates from control HeLa cells (Fig. 4*D*, *lane 3*), when a FLAG-specific antibody was used for Co-IP. As expected, H_2_O_2_ treatment resulted in enhanced SETD2 interactions with MutSα and ATM (Fig. 4*E*).

To determine the effect of the SETD2–ATM and SETD2–MutSα interactions on cellular response to oxidative stress, we expressed the GST-tagged SET domain in *SETD2*^*−/−*^ HeLa cells and measured the protein–protein interactions and ATM phosphorylation by GST pull-down assay after H_2_O_2_ treatment. Both MSH6 and ATM were pulled down in cells expressing the GST-SET domain regardless of H_2_O_2_ treatment (Fig. 4*E*, *lanes 1*, *3*, *5*, and *7*), which further suggests that SETD2 interacts with MutSα and ATM *via* its SET domain. Consistent with the fact that H_2_O_2_ treatment activates ATM in a SETD2/MSH6–dependent manner (Fig. 2*C*), we detected phosphorylated ATM only in H_2_O_2_-treated WT and SET domain–rescued cells (Fig. 4*F*, *lanes 1* and *3*). Taken together, these results suggest that SETD2 forms a complex with MutSα and ATM, which plays a critical role in MMR-mediated DDR signaling in response to oxidative stress.

### MutSα and SETD2 are coenriched in promoter regions in response to oxidative DNA damage

**Figure 5:**
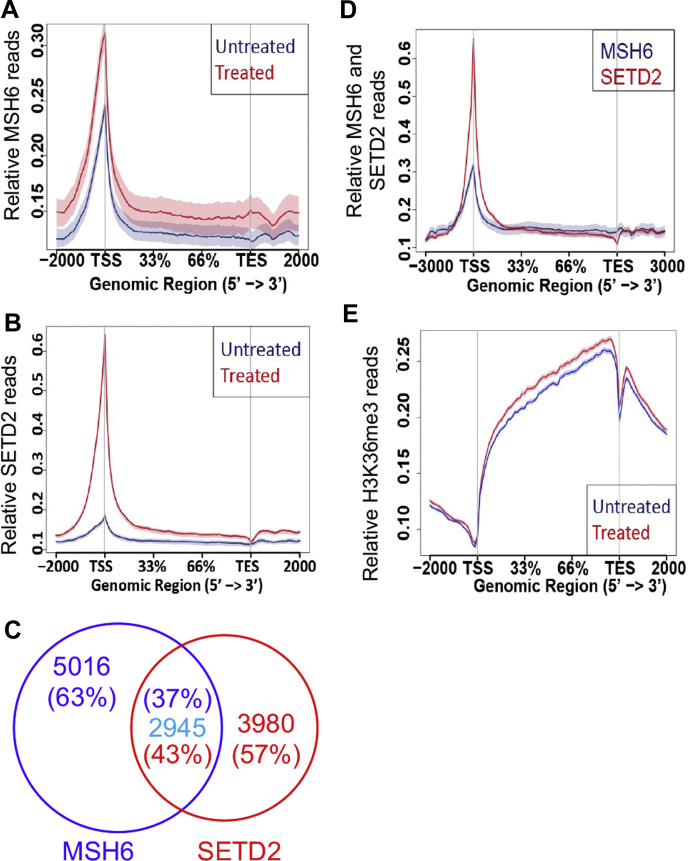
**MutSα and SETD2 are co-enriched in transcription start sites in response to oxidative DNA damage.***A* and *B*, normalized distribution ChIP-Seq profiles of MSH6 (*A*) and SETD2 (*B*) in gene bodies in H_2_O_2_-treated or untreated HeLa FLAG-KI cells. *C*, Venn diagram illustrating the overlap peaks of SETD2 (*red*) and MSH6 (*purple*). *D*, comparison of MSH6 and SETD2 ChIP profiles in H_2_O_2_-treated cells. *E*, normalized distribution profiles of H3K36me3 in gene bodies in H_2_O_2_-treated and untreated cells. ChIP-Seq, chromatin immunoprecipitation assays combined with DNA sequencing; KI, knock-in; TES, transcription end site; TSS, transcription start site.

Previous studies have demonstrated that H3K36me3 and MutSα are colocalized in chromatin (22) and are highly enriched in actively transcribed genes (23, 31). The interaction between MutSα and SETD2 prompted us to hypothesize that MutSα, H3K36me3, and SETD2 are coenriched in chromatin in response to oxidative DNA damage. We therefore performed chromatin immunoprecipitation assays combined with DNA sequencing (ChIP-Seq) analysis to determine the chromatin localization and changes in the abundance of MSH6, SETD2, and H3K36me3 in HeLa cells treated with H_2_O_2_. We found that there were more MSH6 ChIP reads on gene bodies in H_2_O_2_-treated cells than in untreated cells (Fig. 5*A*), which agrees with the Co-IP and chromatin-binding results shown in Figure 1. The SETD2 ChIP-Seq analysis using a FLAG-specific antibody revealed that H_2_O_2_ treatment largely increased the abundance of SETD2 in chromatin in treated cells (Fig. 5*B*). Consistent with the physical interaction between MutSα and SETD2, we observed 2945 overlapping ChIP-Seq peaks between MSH6 and SETD2, accounting for 37% (2945/5016) and 43% (2945/3980) of MSH6 and SETD2 peaks in the genome, respectively (Fig. 5*C*). In addition, MSH6 and SETD2 appeared to be coenriched, peaking at promoter regions/TSSs upon H_2_O_2_ treatment, which suggests that they colocalize and physically interact at TSSs (Fig. 5*D*). Similarly, H_2_O_2_ treatment also stimulated the production of H3K36me3 (Fig. 5*E*). However, unlike with MSH6 and SETD2, we mostly found the enhanced H3K36me3 intensity downstream of promoter regions/TSSs (Fig. 5*E*). The simplest explanation for this phenomenon is that, in the presence of oxidative damage, the preloaded MutSα disassociates from H3K36me3 and binds to a local 8-oxoG adduct at TSSs, which makes the H3K36me3 mark available to recruit a second molecule of MutSα; the 8-oxoG-bound MutSα then interacts with SETD2, which not only recruits ATM to activate DNA damage signaling (see Discussion) but also trimethylates downstream H3K36me2. As a result, both the MutSα and SETD2 levels increase at TSSs, but the H3K36me3 level increases downstream of the damaged TSSs. This assumption is also supported by the fact that MutSα is highly enriched (approximately fourfold) on chromatin in response to H_2_O_2_ treatment (Fig. 1).

## Discussion

Although oxidative DNA lesions are primarily repaired by OGG1-mediated base excision repair, MMR apparently plays an important role in processing oxidative DNA damage. However, the mechanism of the latter is not fully understood. In this study, we show that MMR’s processing of oxidative DNA lesions is a cohesive interaction among MutSα, SETD2, H3K36me3, and ATM, which forms a positive feedback loop during the cellular response to oxidative stress.

As the enzyme that catalyzes the production of H3K36me3, which recruits various DNA repair proteins to chromatin, SETD2 regulates multiple DNA repair pathways, including MMR (22) and double-strand break repair (39, 48, 49). In this study, we show that the MMR-mediated cellular response to oxidative DNA damage requires coordination with SETD2. First, cells depleted of SETD2 accumulate 8-oxoG (Fig. 3*A*) and are more sensitive to H_2_O_2_ treatment than control cells (Fig. 3*C*), phenomena also observed in MMR-deficient cells (Fig. 3, and (14)). Second, like MutSα, SETD2 promotes increased levels of pATM induced by H_2_O_2_ treatment (Fig. 2). Third, MutSα and SETD2 are coenriched in promoter regions/TSSs (Fig. 5). Finally, SETD2 physically interacts with both MutSα and ATM *via* its SET domain (Fig. 4). These observations demonstrate that the MMR-mediated processing of oxidative lesions involves cohesive interactions among MutSα, SETD2, H3K36me3, and ATM. Interestingly, these components, particularly MutSα, SETD2, and H3K36me3, depend on each other for their presence on chromatin. For example, MutSα chromosome localization relies on H3K36me3 (22), which is the product of SETD2 (26). Thus, it is intriguing how these interactions occur and which factor presents first on chromatin.

The data presented in this study seem to have provided an answer for these questions. We found that, in response to H_2_O_2_ treatment, increased MSH6 and SETD2 are coenriched in promoter regions, where genes under active transcription initiation suffer oxidative DNA damage, but the H_2_O_2_-induced increase in H3K36me3 signals is located downstream of promoter regions (Fig. 5). These results suggest that H_2_O_2_-induced recruitment of MSH6 and SETD2 at the initial damage sites has little to do with the H_2_O_2_-induced H3K36me3. Since MutSα specifically recognizes 8-oxoG (11, 12), we believe that the MMR-mediated oxidative response starts with the binding of 8-oxoG lesions by preloaded MutSα, which then recruits SETD2 to the damage site to activate the ATM signaling pathway.

**Figure 6:**
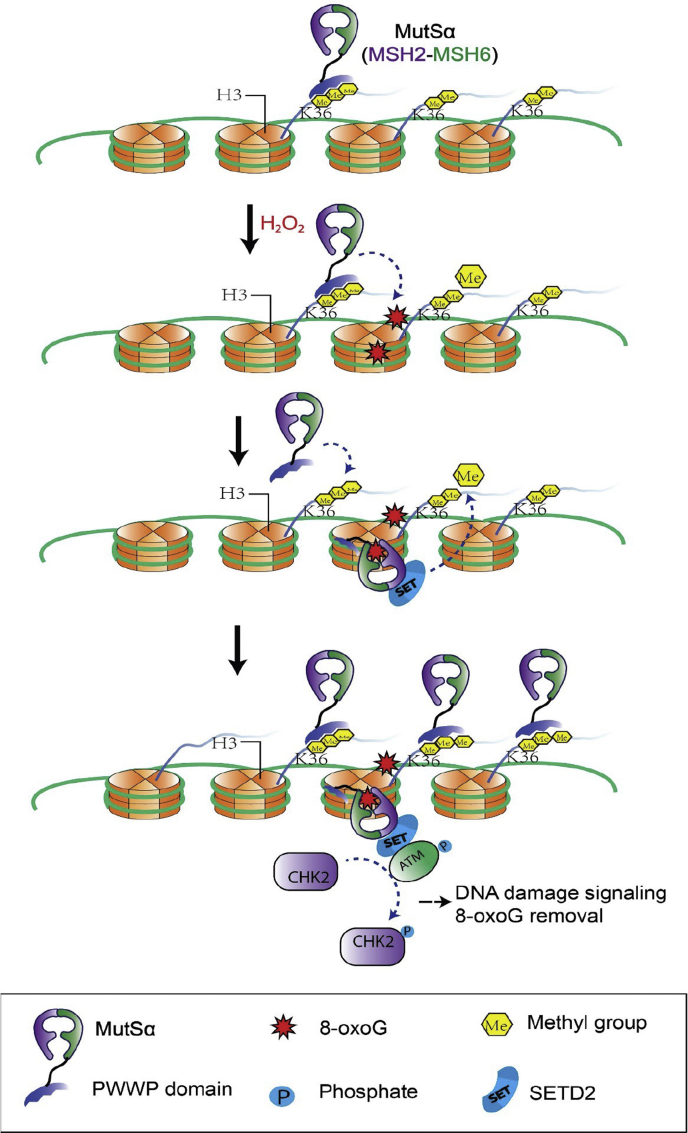
**Proposed model for the MMR-mediated response to oxidative stress.** In the presence of oxidative DNA lesion 8-oxoG, preloaded MutSα binds to a local 8-oxoG adduct. This frees the preoccupied H3K36me3 to recruit additional MutSα, which leads to increased levels of MutSα at damage sites. The 8-oxoG-bound MutSα interacts with SETD2, which in turn recruits ATM to activate the ATM signaling pathway. MutSα-recruited SETD2 trimethylates downstream H3K36, which increases the intensity of H3K36me3 downstream TSSs. The latter can recruit more MutSα molecules to chromatin to respond to further DNA damage. Therefore, in response to oxidative stress, MutSα, SETD2, and H3K36me3 constitute a positive feedback loop to cope with oxidative DNA damage. MMR, mismatch repair; TSS, transcription start site.

Based on previously published data and the results presented here, we propose a working model for the MMR-mediated oxidative response (Fig. 6). In the presence of oxidative DNA lesions such as 8-oxoG, MutSα molecules preloaded by H3K36me3 bind to 8-oxoG lesions, which makes the previously occupied H3K36me3 marks available to recruit additional MutSα molecules to chromatin, which leads to increased levels of MutSα at damage sites. The 8-oxoG-bound MutSα then interacts with SETD2, which in turn recruits ATM, thereby activating the ATM signaling pathway to cope with oxidative DNA lesions. The SETD2 molecules recruited by MutSα catalyze the trimethylation of downstream H3K36, which increases the intensity of H3K36me3 downstream of TSSs. Enriched H3K36me3 can recruit MutSα and other DNA damage/repair factors to maintain genome stability. Therefore, the MMR-mediated oxidative stress response involves a positive feedback loop. This feedback loop starts with MutSα recognizing a lesion, followed by SETD2 coordinating to recruit ATM and trimethylating H3K36, which then loads MutSα onto chromatin for lesion recognition to initiate another feedback loop. However, thorough future studies are required to verify this working model.

## Experimental procedures

### Cell culture

HeLa cells were maintained in Dulbecco’s modified Eagle’s medium (Gibco), and SW620 cells were cultured in RPMI-1640 medium (Gibco) containing 10% fetal bovine serum (HyClone) at 37 °C with 5% CO_2_. HeLa cells stably expressing the SET domain were selected and maintained in medium containing 5 μg/ml puromycin. The CRISPR-Cas9 technology was utilized to generate KO and KI cell lines. For gene KOs, vectors expressing Cas9 and gene-specific single guide RNAs were transfected in HeLa cells (50). For FLAG KI cells, targeting vectors were cotransfected with plasmids expressing Cas9 and specific single guide RNAs. HeLa colonies were picked, expanded, and confirmed by DNA sequencing. Unless mentioned otherwise, cells were treated with 1.0 mM H_2_O_2_ for 30 min, and treated cells were allowed to recover in fresh medium for 24 h before FACS analysis or extract preparation.

### Proteins

The human MutSα protein was expressed and purified as described (21). The complementary DNA–encoding human SETD2 catalytic domain (SET domain, amino acid residues 1418–1714) was cloned into the p-GEX-4T-2 vector (Novagen), expressed in *Escherichia coli*, and purified as a GST-tagged protein. Histone peptide was synthesized as previously described (28).

### Antibodies and inhibitors

The following antibodies were used: anti-phospho-ATM (Ser1981) (CST; 5883), anti-ATM (CST; 2873), anti–phospho-CHK2 (Thr68) (CST; 2197), anti-CHK2 (CST; 6334), anti-MSH6 (BD Biosciences; 610919), anti-MSH2 (CST; 2017), anti-H3K36me3 (Abcam; Ab9050), anti-8-oxoG (Trevigen; 4354-MC-050), anti-FLAG (Sigma; F7425), goat anti-rabbit IgG (Thermo; A10034), goat antimouse IgG (Thermo; A10036), anti-Flag M2 Affinity Gel (Sigma; A2220).

Inhibitors used to block OGG1 and ATM were TH5487 (TOCRIS; 6749) and KU-55933 (Selleck), respectively.

### Immunofluorescence staining

Cells treated with or without H_2_O_2_ were extracted with pre-extraction buffer (0.5% Triton X-100, 1× protease inhibitor cocktail, 10 mM NaF, 10 mM β-glycerophosphate, and 1 mM DTT) for 2 min on ice and fixed by 4% paraformaldehyde. After blocking in 5% bovine serum albumin for 30 min at room temperature (RT), cells were incubated with antibodies (1:100) overnight at 4 °C. After washing with PBS for three times, secondary antibodies were applied for 1 h at RT.

8-oxoG levels were detected per manufacturer’s protocol (Trevigen, 4354-MC-050). Briefly, cells were fixed for 15 min each with −20 °C MeOH and −20 °C acetone, then for 5 min on ice with 0.05 N HCl. After washing with PBS three times, cells were incubated with 100 μg/ml RNase in 150 mM NaCl and 15 mM sodium citrate for 1 h at 37 °C, followed by sequential washing with PBS and 35%, 50%, and 75% EtOH for 4 min each. DNA was denatured *in situ* with 0.15 N NaOH in 70% EtOH for 4 min and washed sequentially with PBS and 70%, 50%, and 35% EtOH containing 4% formaldehyde for 2 min each. Cells were treated with 5 μg/ml proteinase K for 10 min at 37 °C to digest proteins. After blocking with 5% goat serum (in PBS) for 1 h at RT, cells were incubated with an anti-8-oxo-dG antibody at a concentration of 1:250 dilution in PBS containing 1% bovine serum albumin and 0.01% Tween-20 at 4 °C in a humidified chamber overnight. Cells were then washed several times with PBS containing 0.05% Tween-20 before incubating with a fluorescent secondary antibody and mounting with 4′,6-diamidino−2−phenylindole. Slides were analyzed by confocal microscopy (Zeiss 780), and fluorescence intensity was quantified with ImageJ software (National Institutes of Health; https://imagej.nih.gov/ij/download.html).

### Tight chromatin fractionation

Tight chromatin fractionation was performed as previously described (51). Cells were sequentially washed in buffer 1 (10 mM Hepes, pH 7.8, 10 mM KCl, 1.5 mM MgCl_2_, 0.34 M sucrose, 10% glycerol, 0.2% NP-40, 1× protease inhibitor cocktail [Thermo Scientific], and 1× phosphatase inhibitor cocktail [Sigma]), buffer 2 (buffer 1 without NP-40), buffer 3 (3 mM EDTA, 0.2 mM EGTA, and protease inhibitors), and buffer 4 (50 mM Tris–HCl, pH 8.0, 0.05% NP40, 0.45 M NaCl, and protease inhibitors). The remaining pellet was analyzed by Western blot and quantified by ImageJ software.

### Apoptosis analysis

Annexin V staining was used to determine apoptosis according to the manufacturer’s protocol (FITC Annexin V Apoptosis Detection Kit I, BD Biosciences, #556547).

### Immunoprecipitation and GST pull down analyses

For immune pull down assay, the anti-Flag M2 affinity gel, a mouse monoclonal antibody that is covalently attached to agarose, was incubated with cell lysates of FLAG-KI HeLa cells or FLAG-SET transfected HeLa cells in radioimmunoprecipitation assay (RIPA) buffer (50 mM Tris-Cl, pH 8.0, 150 mM NaCl, 1% NP-40, 0.5% NaVO3, and 0.1% SDS) overnight at 4 °C. After washing with RIPA buffer four times to remove nonspecific binding proteins, the gel was subjected to Western blotting analysis to detect proteins pulled down by the FLAG antibody.

GST pull down assay was performed as described (52). Bacterial lysates containing GST fusion proteins were incubated with GST beads for 2 h at 4 °C. After washing with sonication buffer (three times) and RIPA buffer (one time), the fusion protein–containing GST beads were incubated with cell lysate in RIPA buffer. After extensive washing with RIPA buffer, the pull-down proteins were released and analyzed by Western blotting.

### ChIP-Seq analysis

ChIP-Seq analysis was performed as described (23). HeLa and SETD2-KI HeLa cells were treated with or without H_2_O_2_. Cells were fixed with 1% formaldehyde for 10 min, followed by 0.125 M glycine treatment for 5 min. Fixed cells were suspended in cell lysis buffer (50 mM Tri-Cl, pH 8.1, 10 mM EDTA, 1% SDS, 1 mM DTT, 1 mM PMSF, and 1× proteinase inhibitor cocktail) and sonicated to fragment the chromatin. Cell lysates were diluted five times in dilution buffer (16.7 mM Tri-Cl, pH 8.1, 167 mM NaCl, 1.2 mM EDTA, 1.1% Triton X-100, 1 mM PMSF, and 1× proteinase inhibitor cocktail). ChIP-grade antibodies against MSH6, FLAG, or H3K36me3 were incubated with chromatin fragments. Proteins A/G were added to the mixture to pull down the immune complexes, which were washed sequentially with the low salt washing buffer (20 mM Tri-Cl, pH 8.1, 150 mM NaCl, 1 mM EDTA, 1% Triton X-100, 0.1% SDS, and 0.1% Na-deoxycholate), the high salt washing buffer (20 mM Tri-Cl, pH 8.1, 500 mM NaCl, 1 mM EDTA, 1% Triton X-100, 0.1% SDS, and 0.1% Na-deoxycholate), the LiCl washing buffer (10 mM Tri-Cl, pH 8.1, 250 mM LiCl, 1 mM EDTA, 0.5% NP-40, and 0.5% Na-deoxycholate), and Tris-EDTA buffer (10 mM Tris-HCl, pH 8.0, 0.1 mM EDTA). Chromatin was eluted with elution buffer (1% SDS, 50 mM Tris-Cl, 1 mM EDTA, 0.1 M NaHCO3, 250 mM NaCl, and proteinase K), followed by reverse crosslink at 65 °C for 6 h. Purified DNA was used to prepare libraries using NEBNext Ultra II DNA Library Prep kit. Libraries were sequenced on Illumina HiSeq2000. The complete unedited ChIP-Seq datasets are available at NCBI GEO database (accession number GSE163940).

For the analysis of ChIP-Seq data, sequenced reads were mapped to the hg19 genome with default parameters (bowtie2 -x <bt2-idx> -U <r>} [-S <sam>]). The peaks were identified by MACS with the command line “macs14 -t ChIP.bam -c input.bam -f BAM -g h -n test -w --call-subpeaks.” After peak calling, the overlap of peaks across different samples was analyzed by “bedtools intersect” with the criterion of at least one base-pair overlap. The metagene analysis of ChIP-Seq was plotted with the ngs.plot.r algorithm.

## Data availability

All data are contained within the article.

## Conflict of interest

All authors declare that they have no conflicts of interest with the contents of this article.
